# Expression Stabilities of Ten Candidate Reference Genes for RT-qPCR in *Zanthoxylum bungeanum* Maxim

**DOI:** 10.3390/molecules23040802

**Published:** 2018-03-30

**Authors:** Xitong Fei, Qianqian Shi, Tuxi Yang, Zhaoxue Fei, Anzhi Wei

**Affiliations:** College of Forestry, Northwest A&F University, Yangling 712100, China; feixt666@163.com (X.F.); shiqq@nwafu.edu.cn (Q.S.); y2848@126.com (T.Y.); feizhaoxue@163.com (Z.F.)

**Keywords:** *Zanthoxylum bungeanum* Maxim., abiotic stress, stable reference genes, RT-qPCR

## Abstract

Real-time reverse transcription quantitative PCR has become a common method for studying gene expression, however, the optimal selection of stable reference genes is a prerequisite for obtaining accurate quantification of transcript abundance. Suitable reference genes for RT-qPCR have not yet been identified for Chinese prickly ash (*Zanthoxylum bungeanum* Maxim.). Chinese prickly ash is the source of an important food seasoning in China. In recent years, Chinese prickly ash has also been developed as a medicinal plant. The expression stabilities of ten genes (*18S*, *28S*, *EF*, *UBA*, *UBQ*, *TIF*, *NTB*, *TUA*, *RPS*, and *TIF5A*) were evaluated in roots, stems, leaves, flowers and fruits at five developmental stages and also under stress from cold, drought, and salt. To do this we used three different statistical algorithms: geNorm, NormFinder and BestKeeper. Among the genes investigated, *UBA* and *UBQ* were found to be most stable for the different cultivars and different tissues examined, *UBQ* and *TIF* for fruit developmental stage. Meanwhile, *EF* and *TUA* were most stable under cold treatment, *EF* and *UBQ* under drought treatment and *NTB* and *RPS* under salt treatment. *UBA* and *UBQ* for all samples evaluated were most stably expressed, but *18S*, *TUA* and *RPS* were found to be generally unreliable as reference genes. Our results provide a basis for the future selection of reference genes for biological research with Chinese prickly ash, under a variety of conditions.

## 1. Introduction

The genus *Zanthoxylum* belongs to the family Rutaceae and has a long history of cultivation in China where it is valued both as a food plant and also for its traditional medicinal properties [1]. The species *Z. bungeanum* Maxim. (ZBM) is native to eastern China and is used primarily as a peppery spice [2]. This is one of the eight main spices used in China and is an essential ingredient for hot pots [3]. A number of pharmacological studies have shown that ZBM extracts can also be used in the treatment of inflammatory diseases including ascariosis, diarrhea and dysentery [4]. 

Popularly known as ‘Chinese prickly ash’, the widely-cultivated ZBM includes two cultivars, Green Huajiao and Red Huajiao—thus named for the color of their fruits. These are especially used as a spice in indigenous kitchens in China [5]. The most important production centers for Chinese prickly ash are Shaanxi, Gansu and Sichuan provinces.

The study of reference gene selection for Chinese prickly ash can provide a basis for the quantitative analysis and for qualitative analysis of the related plant species. Housekeeping genes, such as *Actin-depolymerizing factor* (*ACT*), *glyceraldehyde-3-phosphate dehydrogenase* (*GAPDH*), *α-Tubulin* (*TUB*) [6] and *elongation factor 1-alpha* (*EF*), have often been used as reference genes [7]. The most common method for measuring mRNA expression level is reverse transcription quantitative real-time PCR (RT-qPCR). To obtain optimal evaluations of RT-qPCR data it is essential that the expression of the reference genes be stable [8]. Hence, reference gene expression level must be stable under a diversity of conditions. These conditions should include: at a range of stages of organ development, across a range of different plant tissues and under exposure to as wide a range as possible of stress conditions. Last, reference gene selection is critical for normalization. For plants, a large number of genes have been proposed as exhibiting sufficient stability to render them suitable as reference genes [9,10,11]. Choosing reliable reference genes as internal controls to normalize gene expression in qRT-PCR is extremely important for reducing errors and for determining accurate expressions of target genes [12].

In the present study with ZBM, the expression stabilities of 10 candidate reference genes were evaluated under a range of conditions and treatments. BestKeeper, geNorm and NormFinder were used to analyze the expression stabilities of these genes. The results not only identify useful reference gene resources in the genus *Zanthoxylum* but also offer a guide for related research with other plant species.

## 2. Results

**Figure 1:**
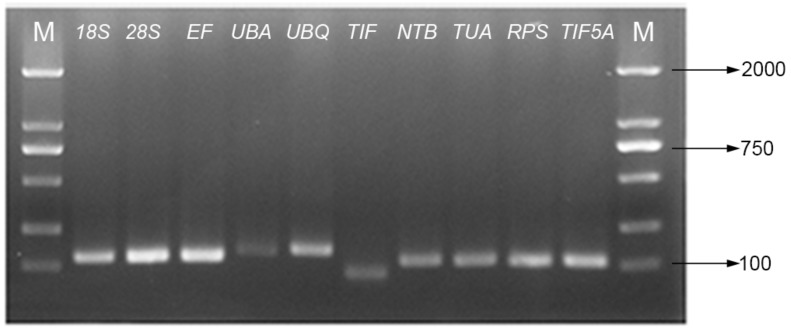
Agarose gel analysis of primers specificities in amplification of candidate genes.

**Figure 2:**
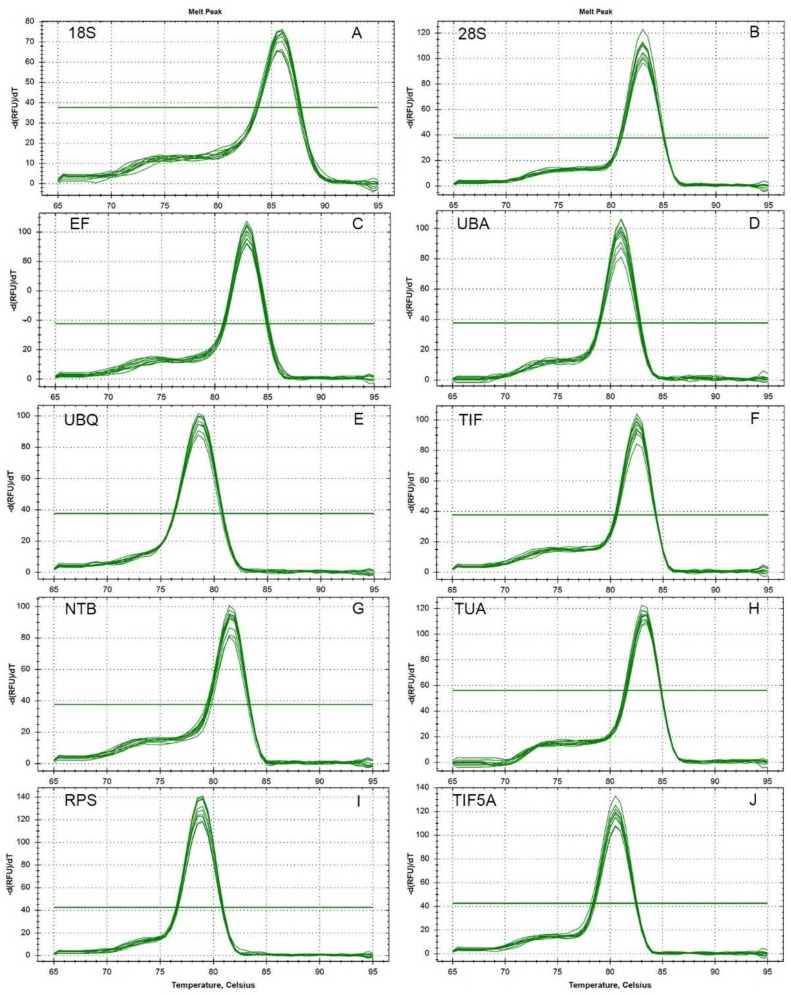
Melt curves of the 10 reference genes, *18S*, *28S*, *EF*, *UBA*, *UBQ*, *TIF*, *NTB*, *TUA*, *RPS*, and *TIF5A*.

Ten candidate reference genes (*18S*, *28S*, *EF*, *UBA*, *UBQ*, *TIF*, *NTB*, *TUA*, *RPS*, and *TIF5A*) were assessed to normalize their expression stabilities in Chinese prickly ash using RT-qPCR. Cycle threshold values (Ct) were used to determine the expression levels of the 10 candidate reference genes in all samples. The expression stabilities of the ten candidate reference genes were analyzed using geNorm (version 3.5 Applied Biosystems, Foster City, CA, USA), NormFinder (version 0.953, Foster City, CA, USA) and BestKeeper (version 1, Foster City, CA, USA). A total of 10 candidate reference genes representing different classes were selected for the experiment. The gene symbols, descriptions, primer sequences, amplicon sizes, GeneBank accession numbers, amplification efficiencies (E) and correlation coefficients (R^2^) are listed in Table 1. After amplification of pooled cDNA, all primer pairs of the 10 reference genes yielded a single PCR product of the expected size. There was no evidence of primer dimer formation or of non-specific amplification (Figure 1). Meanwhile, the presence of single peaks in the melt curve analyses confirmed the specificity of amplicons and also indicated the melting temperatures (Figure 2). Among the genes investigated, *UBA* and *UBQ* were found to be most stable for the different cultivars and different tissues examined, *UBQ* and *TIF* for fruit developmental stage. Meanwhile, *EF* and *TUA* were most stable under cold treatment, *EF* and *UBQ* under drought treatment and *NTB* and *RPS* under salt treatment. *UBA* and *UBQ* for all samples evaluated were most stably expressed, but *18S*, *TUA* and *RPS* were found to be generally unreliable as reference genes.

After 40 cycles of amplification, a melting curve analysis of each primer set was carried out using RT-qPCR. Single peaks indicate the expected amplicons were detected with SYBR Green. As indicated by the agarose gel electrophoreses, each of the ten primer pairs amplified a single band of the expected size from the various cDNA templates. The correlation coefficients (R^2^) ranged from 0.992 to 0.999 and the PCR amplification efficiencies between 92.8 and 108.4% (The theoretical range is 90% to 110%) were obtained from the standard curves generated using a five-fold serial dilution of cDNA.

The Ct values were monitored under seven conditions. These included: all samples; different cultivars; different tissues; different developmental stages of fruits; with/without cold stress; with/without drought stress and with/without salt stress. The mean Ct values of the 10 potential reference genes ranged from 21.29 to 33.58 (Table 2). In all tested samples, the mean Ct values showed a minimum of 21.29 ± 0.79 and a maximum of 31.48 ± 1.00 for the highest and lowest expression levels for *18S* and *TIF*, respectively. *18S* and *28S* also showed minimum and maximum average Ct values, respectively, in all experimental groups, while UBA had maximum average Ct value of 33.58 ± 0.35.

The coefficient of variation (CV) of the Ct values was used to evaluate the expression levels of candidate reference genes in the experiments. Low CV values indicate low variability (i.e., high stability) [12]. The CV of the 10 reference genes among all samples ranged between 0.55 and 8.61%. *NTB* was the least variable reference gene with a CV of 0.55% among the 10 candidate reference genes studied, *RPS* was the most variable with a CV of 8.61%. On the basis of the CV values, the stability ranking of all candidate reference genes was: *TIF* < *NTB* < *EF*< *TIF5A* < *28S* < *UBQ* < *UBA* < *18S* < *RPS* < *TUA* (Table 2).

### 2.1. geNorm Analysis

To evaluate gene expression stability, the geNorm analysis uses the reference gene expression stability measurement (M) value (calculated as the level of pairwise variation for each reference gene with all other control genes and as the SD of the logarithmically transformed expression ratios). High M values indicate low stability [13].

**Figure 3:**
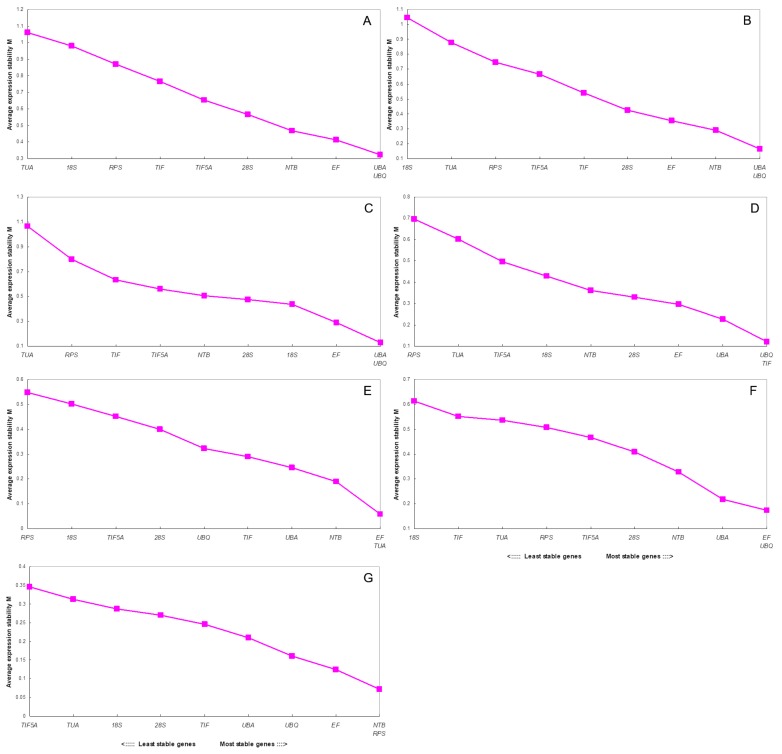
Average expression stability values (M) of 10 candidate reference genes (*18S, 28S, EF, UBA, UBQ, TIF, NTB, TUA, RPS*, and *TIF5A*) by GeNorm analysis:(**A**) all samples; (**B**) different cultivars; (**C**) different tissues; (**D**) different developmental stages of fruits; (**E**) under cold stress; (**F**) under drought stress and (**G**) under salt stress.

The plants were exposed to a range of treatments to generate data for the geNorm analysis. The Cq values were processed linearly using the ΔCq method. Next, the Cq values were converted to relative quantity values using the formula 2 − ΔCq (where ΔCq is Cq minus the minimum Cq value). The various reference genes had a range of different stabilities. geNorm calculates the value of Vn/n + 1 between normalization factors, which is then used to determine the number of reference genes required for optimal normalization. Vn/n + 1 values < 0.15 indicates that the introduction of additional genes will not contribute significantly to normalization [13]. The V2/3 values for total (0.146), different cultivars (0.116), different tissues (0.121), different fruits (0.090), cold treatments (0.084), drought treatments (0.075) and salt treatments (0.050) were all lower than 0.15 (Figure 3). This indicates two reference genes are sufficient for accurate normalization of all samples. 

**Figure 4:**
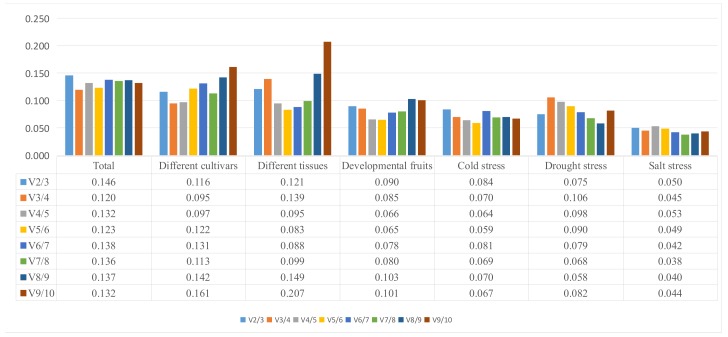
Determination of best reference gene number calculated by geNorm pairwise variation (Vn/Vn + 1).

In different cultivars, the most stable genes were *UBA* and *UBQ*. The M value obtained for these two genes was 0.165 (Figure 3B), and the V value was 0.116 (Figure 4). Meanwhile, in different tissues, these two genes also showed stability, M and V were 0.126 (Figure 3C) and 0.121 (Figure 4), respectively. In the cold treatments, *EF* and *TUA* were identified as the most stable genes, with an M value of 0.059 (Figure 3E) and V value of 0.084 (Figure 4). GeNorm indicates *EF* and *UBQ* as reliable reference genes in the drought stress treatment. Here, the M and V values of these two genes were 0.173 (Figure 3F) and 0.075 (Figure 4), respectively.

For fruits of different developmental stages, *UBQ* and *TIF* were considered the most stable genes, with an M value of 0.120 (Figure 3D) and a V value of 0.090 (Figure 4). In the salt-stress treatment, *NTB* and *RPS* were identified as the most stable genes in geNorm, with an M value of 0.073 (Figure 3G) and a V value of 0.050 (Figure 4). In the total materials, *UBA* and *UBQ* were the best reference genes, again according to geNorm, the M value obtained for these two genes was 0.323 (Figure 3A) and the V value was 0.146 (Figure 4). For different tissues, the most stable genes were *EF* and *18S* but geNorm indicates *18S*, *TUA* and *RPS* as unreliable reference genes for most experiments. More interestingly, the same reference genes seem to have different stabilities under different treatments. Thus, *RPS* was the most stable gene for salt stress and the least stable for cold stress.

Table 3 shows the results for all samples based on geNorm. The M values of *EF* (0.790) and *UBQ* (0.819) were <1.5. These were the lowest values from the gene expression analyses for all samples. In contrast, *UBQ* (0.731) and *UBA* (0.764) were the most stable genes among cultivars (i.e., having the lowest M values). In different tissues, *EF* (0.764) and *18S* performed well in terms of gene expression stability. These results indicate that *28S* (0.31) and *UBQ* (0.536) would be suitable for normalizing gene expression in developing fruits. For abiotic stress, gene expression stability was very different. We found *TUA* (0.407), *TIF5A* (0.507) and *RPS* (0.267) were the most stable genes under low-temperature stress, drought stress and salt stress, respectively.

### 2.2. NormFinder Analysis

**Figure 5:**
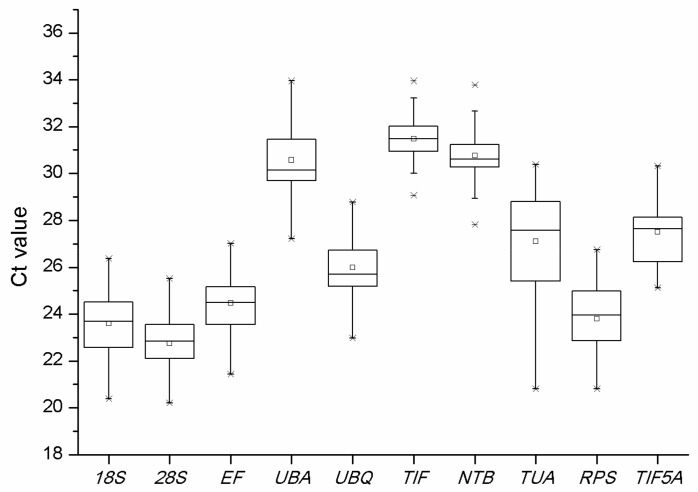
Distribution of the Cq-values of the ten candidate reference genes (*18S, 28S, EF, UBA, UBQ, TIF, NTB, TUA, RPS*, and *TIF5A*) across all samples in qPCR analyses.

NormFinder was used to identify the optimal normalization gene for any particular experimental design. As with geNorm, the data from a qRT-PCR should first be transformed [13]. The gene stability rankings are shown in Table 4. Distribution of the Cq-values of the ten candidate reference genes across all samples in qPCR analyses (Figure 5).

According to the NormFinder analysis, the two most stable reference genes for all samples (Total) were *UBA* (0.001) and *NTB* (0.001), while *UBA* and *NTB* were the most stable genes for different cultivars. The two most stable reference genes for different tissues samples were *TIF5A* and *UBA*. In different fruits samples, *UBA* and *TIF* ranked as the most stable reference genes, while *RPS* was the least stable reference gene in the group. *UBA* and *TUA* were considered the most stable reference genes by NormFinder in the cold treatments. *TIF5A* and *UBQ* were each ranked first for stability in the drought treatments and in the salt treatments, respectively.

However, in most samples, the stability ranking of candidate reference genes created with NormFinder was slightly different from that with GeNorm. For example, *UBA* and *NTB* were identified as the most stable reference genes for all samples and different cultivars in the NormFinder analysis but the stability rankings of *UBA* and *UBQ* were identified as the most stable with the GeNorm analysis.

### 2.3. BestKeeper Analysis

BestKeeper is an Excel™-based tool that uses pairwise correlation: to determine the stability of housekeeping genes, to identify differentially regulated target genes and to confirm sample integrity. BestKeeper can be used to analyze the stability of candidate reference genes, based on the CV and SD of the Cq values. It uses the average Cq value of each duplicate reaction [14].

The CV and the standard deviation (SD) of the candidate reference genes were used to evaluate reference gene stability in each experiment. Here, genes with low CV and SD values are the most stable [15]. The most stable reference genes present the lowest CV and SD (CV ± SD). Values of SD of less than 1 are considered an acceptable range of variation [16].

BestKeeper differs from the geNorm and the NormFinder analyses in that it can take as input for the analysis the raw Cq values. As with the NormFinder results, the CV ± SD rankings of the candidate genes increase from top to bottom, indicating a gradual stability decrease. For instance, *NTB* had a CV ± SD value of 0.55 ± 0.17 and is ranked as the most stable gene in drought stress, while, 18S is listed as the least stable gene, with a CV ± SD value of 2.22 ± 0.56 (Table 5).

## 3. Discussion

In general, an ideal reference gene is one that is stably expressed under a very wide range of experimental conditions and among a wide range of tissues. In this study, no one reference gene was consistently expressed across all samples evaluated. For example, *UBA* and *UBQ* were suitable as reference genes among different cultivars, different tissues and all samples, but these two genes were not stably expressed under abiotic stresses, like *EF* and *TUA* for the cold treatment, *EF* and *UBQ* for the drought treatment, *NTB* and *RPS* for the salt treatment. Meanwhile, *UBQ* and *TIF* were stably expressed among the fruit developmental stages.

From comparative analyses of previously published studies of reference gene identification for fruit developmental in other plants, we found two reference genes in this study had previously been chosen as optimal reference genes for fruit developmental in other plants. In bamboo, *UBQ* is the most stable among a range of tissues, while *NTB* is suitable among a range of tissues and over a range of developmental stages [17]. Our study draws similar conclusions, *UBA* and *UBQ* for all samples evaluated were stably expressed, and *NTB* is also very stable in the cold treatment. Abiotic stresses, such as drought, salinity and extreme temperatures, always limit plant growth and yield [11]. In our study, expression levels of the 10 candidate genes all decreased under the various abiotic stresses and, hence, the stability of gene expression reduced. Among different cultivars, *UBA* is the most stable gene but under drought or salt stresses, *UBA* is only the sixth and the fifth most stable gene, respectively. In this study, geNorm, NormFinder and BestKeeper were adopted to evaluate the stability of gene expression, and the results showed some difference among the three methods. If a candidated gene showed good stability under two of the methods, it was considered as stable inference gene.

A few genes have been used repeatedly as reference genes in a range of plant species. These include *EF*, *UBC*, *EXP*, *GAPDH* and *F-box protein* [6,7,18,19,20,21]. Recent studies suggest these genes are not always expressed stably in other species or under a different experimental conditions [7]. For example, *EXP* and *UBQ* have been shown to perform poorly and be less stable under ABA treatment [2]. *GADPH* and *F-box protein* have not been considered suitable reference genes for RT-qPCR data normalization [22].

In the roots, stems, leaves, flowers and seeds of mature plants, *elongation factor-1alpha* (*EF*) has been suggested as a useful reference gene for RT-qPCR in *Plukenetia volubilis* L. [23]. However, in this study, this gene did not appear to be the best, as some variation in expression appeared among the treatments. In *Descurainia sophia*, *18S* was the most stable reference gene in all samples tested [24]. This gene has also been used as the reference gene for target gene expression analysis in papaya under a range of experimental conditions [25]. Nevertheless, in our study, the *18S* gene with M of 1.1381 did not prove a good reference gene for RT-qPCR data normalization.

Through comparative analyses of our experimental results, the performance of these housekeeping genes among different species is very variable, and is also variable in the same species but under different experimental treatments. The results presented here not only identify optimal reference genes for qPCR analysis in *Zanthoxylum bungeanum*, but also offer guidelines for identification of reference genes in other plant species.

## 4. Materials and Methods

### 4.1. Materials

#### 4.1.1. Plant Materials

Leaves of five cultivars of *Z. bungeanum* were collected—Fengxiandahongpao, Fuguhuajiao, Hanchengdahongpao, Xinongwuci and Hanyuanhuajiao. Fruits of Fengxiandahongpao were collected at five developmental stages—young fruit, enlarging fruit, green mature fruit, half-red fruit and full-red fruit. Five organs/tissues of “Fengxiandahongpao” were collected from three trees including: roots, stems, leaves, flowers and fruits. In addition, three kinds of stress treatment were imposed on carried out with one-year-old Chinese Prickly ash seedlings, which were salt stress, drought stress and cold stress. Each sampling was repeated three times.

Materials were harvested from the Experimental Station of *Zanthoxylum bungeanum*, Northwest A&F University in Fengxian, Shaanxi, China. The fruit samples were quickly shelled and cut into small pieces, these were immersed in liquid nitrogen and stored at −80 °C. The leaves were frozen directly in liquid nitrogen and then stored at −80 °C pending RNA extraction.

#### 4.1.2. Stress Treatments

Uniform, one-year-old Chinese Prickly ash seedlings were used as experimental material. The ZBM plants were subjected to three abiotic stress treatments: (1) salt stress, (2) drought stress and (3) cold stress. For salt stress plants were transferred from 50% *Murashige* and *Skoog* medium (½MS) to fresh ½MS supplemented with 1.2% NaCl. For drought stress, drought was simulated using 15% PEG 6000 added to ½MS. For cold stress: roots were watered with ½MS in daylight and then placed in a 4 °C controlled temperature environment. The samples of all treatments were collected after 0, 4, 24 and 48 h.

### 4.2. Methods

#### 4.2.1. Total RNA Extraction and cDNA Synthesis

Total RNA was extracted from all samples and purified using the TaKaRa MiniBEST Plant RNA Extraction Kit (TaKaRa, Beijing, China) according to the manufacturer’s instructions. Total RNA purity and concentration were determined using a NanoDrop 20000 (Thermo Scientific, Pittsburgh, PA, USA). Only RNA samples with OD260/280 ratios between 1.8 and 2.2, and OD260/230 ratios higher than 2.0 were used for cDNA synthesis.

#### 4.2.2. Primer Design and qPCR

The candidate genes were selected from the research of the related families and some traditional reference genes [6]. Based on the genome sequencing of *Citrus sinensis* [26] and *Zanthoxylum* transcriptional data, 10 candidate reference genes were selected (*18S, 28S, EF, UBA, UBQ, TIF, NTB, TUA, RPS*, and *TIF5A*). The sequences were downloaded from NCBI and then compared on the NCBI website, selecting the conserved sequence for primer design. Ten pairs of primers were designed using Primer Premier 5.0 (Palo Alto, CA, USA).

The sequences of all primers used in study are listed in Table 1. To test the stability of reference gene expression, qRT-PCR was used to measure the transcript levels. The qRT-PCR assays were carried out on a CFX96 Real-Time PCR Detection System (Bio-Rad, Hercules, CA, USA). A standard curve was produced using serial dilutions of a cDNA from *Zanthoxylum bungeanum*. This was used to evaluate the efficiency of each primer set. Each 10 μL reaction used 5 μL of 2× SYBR Premix Ex Taq Ⅱ (TaKaRa), 1 μL of cDNA, 0.5 μL of each primer with a final concentration of 1 μM, and 3 μL ddH2O. qRT-PCR amplifications were carried out using the program: 95 °C for 30 s followed by 40 cycles of 94 °C for 5 s, 54 °C for 30 s, and 72 °C for 45 s. The PCR products were then analyzed on 1% (*m/v*) agarose gel.

## 5. Conclusions

In this study, ten candidate reference genes were selected, to evaluate their expression stabilities by qRT-PCR under three abiotic treatments, among different plant tissues, between different cultivars and among fruits at different stages of development. To do this, we used the geNorm, NormFinder and BestKeeper statistical algorithms. We show that the following genes were stably expressed: UBA and UBQ for the different cultivars, UBA and UBQ for the different tissues, UBQ and TIF for fruit development, EF and TUA for the cold treatment, EF and UBQ for the drought treatment, NTB and RPS for the salt treatment and UBA and UBQ for all samples.

## Figures and Tables

**Table 1:** Primer and related information for the 10 candidate reference genes *18S*, *28S*, *EF*, *UBA*, *UBQ*, *TIF*, *NTB*, *TUA*, *RPS*, and *TIF5A* for quantitative RT-PCR analysis in *Zanthoxylum bungeanum* Maxim.

Gene Symbol	Description	Primer Sequence (5′-3′)		GeneBank Accession Number	Amplicon Size	E(%)	R^2^
*18S*	18S ribosomal RNA gene	F-GCGGATTGTGCCAAGGAA	R-ATATCCGTTGCCGAGAGTCG	KC502933.1	100	98.9	0.998
*28S*	28S ribosomal RNA gene	F-GTCGCCTTCTTTCGCTCTGTC	R-GGTTCACGGGATTCTGCAATT	HM851494.1	118	105.8	0.999
*EF*	Elongation factor 1-alpha	F-GTGCTTGACTGCCACACCTC	R-TTCCGGCATCTCCATTCTTC	XM_006488084.2	107	92.8	0.993
*UBA*	ubiquitin-60S ribosomal protein L40	F-GACTTAGGGGAGGGATTATTGAG	R-TTCTTCTTCCGACAGTTTACAGC	XM_006481681.2	123	96.7	0.998
*UBQ*	ubiquitin extension protein	F-TCGAAGATGGCCGTACATTG	R-TCCTCTAAGCCTCAGCACCA	AB906612.1	122	95.7	0.996
*TIF*	Translation initiation factor	F-TTCCTCCCATTACGTTGCT	R-GCTGGTTACGGACTCTTTG	JK724818.1	165	93.4	0.987
*NTB*	Nucleotide tract-binding protein	F-CTTTGGACGGGAGAAGTAT	R-TCACAGGAAGATAGGGATTT	XM_006482555.2	150	98.4	0.999
*TUA*	tubulin	F-GTAGGCGGAGGAGATGATGC	R-GATGGAAGAGTTGGCGGTAAGT	GU911362.1	142	108.4	0.992
*RPS*	ribosomal protein S16	F-GAAATCCAAAAGCAAGGGG	R-AAAATGGCAGCAACACACC	KJ364714.1	135	92.8	0.998
*TIF5A*	translation initiation factor 5A	F-ATATTGTGCCTTCCTCCC	R-GCCTCAGATCATCCTTGG	XM_006473932.1	122	94.8	0.995

**Table 2:** Ct values of the ten reference genes (*18S*, *28S*, *EF*, *UBA*, *UBQ*, *TIF*, *NTB*, *TUA*, *RPS*, and *TIF5A*) as calculated for *Zanthoxylum bungeanum*.

Genes	Total (Ct ± SD)	Different Cultivars (Ct ± SD)	Different Tissues (Ct ± SD)	Developmental Fruits (Ct ± SD)	Cold Stress (Ct ± SD)	Drought Stress (Ct ± SD)	Salt Stress (Ct ± SD)
*18S*	23.61 ± 1.52	23.14 ± 1.79	22.79 ± 1.82	23.25 ± 0.71	24.59 ± 1.03	25.31 ± 0.90	24.16 ± 0.53
*28S*	22.76 ± 1.21	21.53 ± 0.59	21.40 ± 0.62	22.86 ± 0.25	24.22 ± 0.89	23.18 ± 0.60	23.77 ± 0.51
*EF*	24.46 ± 1.24	23.24 ± 0.26	23.20 ± 0.24	24.57 ± 0.49	26.49 ± 0.37	24.83 ± 0.56	24.71 ± 0.50
*UBA*	30.58 ± 1.70	28.88 ± 0.62	28.81 ± 0.58	30.85 ± 0.66	33.58 ± 0.35	30.86 ± 0.70	30.63 ± 0.78
*UBQ*	25.99 ± 1.45	24.58 ± 0.55	24.48 ± 0.55	26.10 ± 0.49	28.54 ± 0.24	26.30 ± 0.48	26.08 ± 0.52
*TIF*	31.48 ± 1.00	31.49 ± 0.57	31.49 ± 0.51	31.41 ± 0.50	33.07 ± 0.67	31.40 ± 0.75	30.77 ± 0.81
*NTB*	30.76 ± 1.22	29.77 ± 0.68	29.75 ± 0.61	31.01 ± 0.44	32.90 ± 0.60	30.71 ± 0.20	30.91 ± 0.51
*TUA*	27.11 ± 2.12	25.67 ± 1.50	25.53 ± 1.38	26.45 ± 0.66	29.78 ± 0.41	28.04 ± 0.53	28.32 ± 0.44
*RPS*	23.8 ± 1.67	21.39 ± 0.84	21.29 ± 0.79	24.13 ± 0.95	25.76 ± 0.95	23.75 ± 0.29	24.55 ± 0.56
*TIF5A*	27.5 ± 1.42	25.88 ± 0.63	25.8 ± 0.61	27.08 ± 0.58	29.69 ± 0.65	27.72 ± 0.47	28.44 ± 0.84

**Table 3:** Ranking of the candidate reference genes according to their stability value using geNorm.

Rank	Total		Different Cultivars		Different Tissues		Developmental Fruits		Cold Stress		Drought Stress		Salt Stress	
	Gene	M	Gene	M	Gene	M	Gene	M	Gene	M	Gene	M	Gene	M
1	*EF*	0.790	*UBQ*	0.731	*EF*	0.763	*28S*	0.531	*TUA*	0.407	*TIF5A*	0.507	*RPS*	0.267
2	*UBQ*	0.819	*UBA*	0.764	*18S*	0.794	*UBQ*	0.536	*EF*	0.418	*28S*	0.512	*NTB*	0.274
3	*NTB*	0.867	*EF*	0.772	*UBA*	0.809	*TIF*	0.554	*NTB*	0.434	*UBQ*	0.568	*UBQ*	0.285
4	*28S*	0.895	*28S*	0.817	*UBQ*	0.818	*EF*	0.559	*UBA*	0.544	*NTB*	0.574	*EF*	0.315
5	*UBA*	0.937	*NTB*	0.851	*28S*	0.834	*UBA*	0.610	*28S*	0.544	*TUA*	0.584	*28S*	0.341
6	*TIF5A*	0.994	*TIF*	1.058	*NTB*	0.887	*NTB*	0.633	*TIF*	0.558	*RPS*	0.587	*UBA*	0.343
7	*TIF*	1.229	*TIF5A*	1.140	*TIF5A*	0.955	*TIF5A*	0.711	*TIF5A*	0.585	*TIF*	0.606	*18S*	0.365
8	*RPS*	1.315	*RPS*	1.204	*TIF*	1.134	*18S*	0.807	*UBQ*	0.591	*EF*	0.662	*TIF*	0.384
9	*18S*	1.381	*TUA*	1.397	*RPS*	1.513	*TUA*	0.952	*18S*	0.670	*UBA*	0.674	*TUA*	0.408
10	*TUA*	1.395	*18S*	1.706	*TUA*	2.132	*RPS*	1.077	*RPS*	0.736	*18S*	0.865	*TIF5A*	0.485

**Table 4:** Stability analysis of candidate reference genes, as assayed with NormFinder software.

Rank	Total		Different Cultivars		Different Tissues		Developmental Fruits		Cold Stress		Drought Stress		Salt Stress	
	Gene	Stability Value	Gene	Stability Value	Gene	Stability Value	Gene	Stability Value	Gene	Stability Value	Gene	Stability Value	Gene	Stability Value
1	*UBA*	0.001	*UBA*	0.000	*TIF5A*	0.005	*UBA*	0.000	*UBA*	0.003	*TIF5A*	0.003	*UBQ*	0.004
2	*NTB*	0.001	*NTB*	0.000	*UBA*	0.017	*TIF*	0.000	*TUA*	0.003	*TUA*	0.005	*TIF5A*	0.005
3	*TIF*	0.001	*TIF*	0.001	*UBQ*	0.018	*NTB*	0.001	*TIF5A*	0.003	*NTB*	0.021	*TUA*	0.015
4	*TIF5A*	0.008	*TUA*	0.013	*NTB*	0.022	*28S*	0.006	*NTB*	0.004	*TIF*	0.024	*NTB*	0.023
5	*UBQ*	0.025	*UBQ*	0.014	*TIF*	0.023	*UBQ*	0.006	*28S*	0.004	*28S*	0.024	*UBA*	0.023
6	*EF*	0.112	*TIF5A*	0.024	*28S*	0.023	*TIF5A*	0.011	*TIF*	0.005	*UBA*	0.024	*TIF*	0.025
7	*TUA*	0.119	*EF*	0.025	*EF*	0.079	*EF*	0.056	*UBQ*	0.025	*UBQ*	0.031	*28S*	0.026
8	*28S*	0.124	*28S*	0.243	*18S*	0.127	*TUA*	0.059	*EF*	0.091	*EF*	0.129	*18S*	0.069
9	*18S*	0.309	*RPS*	0.333	*TUA*	0.269	*18S*	0.269	*18S*	0.117	*18S*	0.143	*EF*	0.113
10	*RPS*	0.338	*18S*	0.403	*RPS*	0.376	*RPS*	0.318	*RPS*	0.127	*RPS*	0.210	*RPS*	0.117

**Table 5:** Expression stability values of the ten candidate reference genes (*18S*, *28S, EF, UBA, UBQ, TIF, NTB, TUA, RPS*, and *TIF5A*) calculated using BestKeeper.

Rank	Total		Different Cultivars		Different Tissues		Developmental Fruits		Cold Stress		Drought Stress		SALT STRESS	
	Gene	CV ± SD	Gene	CV ± SD	Gene	CV ± SD	Gene	CV ± SD	Gene	CV ± SD	Gene	CV ± SD	Gene	CV ± SD
1	*TIF*	2.30 ± 0.72	*EF*	0.92 ± 0.21	*TIF*	2.41 ± 0.74	*28S*	0.89 ± 0.20	*UBQ*	0.59 ± 0.17	*NTB*	0.55 ± 0.17	*TUA*	1.00 ± 0.28
2	*NTB*	2.77 ± 0.85	*TIF*	1.19 ± 0.37	*NTB*	2.65 ± 0.79	*NTB*	1.08 ± 0.33	*UBA*	0.80 ± 0.27	*RPS*	0.91 ± 0.22	*NTB*	1.27 ± 0.39
3	*EF*	3.90 ± 0.95	*UBQ*	1.48 ± 0.36	*28S*	3.98 ± 0.86	*TIF*	1.30 ± 0.41	*TUA*	1.01 ± 0.30	*UBQ*	1.26 ± 0.33	*EF*	1.56 ± 0.39
4	*TIF5A*	3.96 ± 1.09	*UBA*	1.50 ± 0.43	*TIF5A*	4.04 ± 1.09	*UBQ*	1.51 ± 0.40	*EF*	1.04 ± 0.27	*TUA*	1.35 ± 0.38	*UBQ*	1.71 ± 0.45
5	*28S*	4.16 ± 0.91	*NTB*	1.56 ± 0.47	*UBA*	4.13 ± 1.21	*TIF5A*	1.58 ± 0.43	*NTB*	1.33 ± 0.44	*TIF5A*	1.46 ± 0.40	*18S*	1.77 ± 0.43
6	*UBQ*	4.29 ± 1.12	*TIF5A*	2.00 ± 0.52	*18S*	4.16 ± 0.91	*EF*	1.60 ± 0.39	*TIF*	1.57 ± 0.52	*EF*	1.47 ± 0.36	*RPS*	1.78 ± 0.44
7	*UBA*	4.38 ± 1.34	*28S*	2.20 ± 0.47	*EF*	4.21 ± 0.99	*TUA*	1.69 ± 0.45	*TIF5A*	1.74 ± 0.52	*UBA*	1.64 ± 0.51	*28S*	1.82 ± 0.43
8	*18S*	5.06 ± 1.19	*RPS*	2.76 ± 0.59	*UBQ*	4.62 ± 1.15	*UBA*	1.84 ± 0.57	*28S*	2.70 ± 0.66	*28S*	2.01 ± 0.47	*UBA*	2.09 ± 0.64
9	*RPS*	5.46 ± 1.30	*TUA*	4.67 ± 1.20	*RPS*	6.52 ± 1.55	*18S*	2.53 ± 0.59	*RPS*	2.72 ± 0.70	*TIF*	2.04 ± 0.64	*TIF5A*	2.10 ± 0.60
10	*TUA*	6.06 ± 1.64	*18S*	6.41 ± 1.48	*TUA*	8.61 ± 2.19	*RPS*	3.31 ± 0.80	*18S*	2.98 ± 0.73	*18S*	2.22 ± 0.56	*TIF*	2.26 ± 0.69
